# Tools for the *ex situ* conservation of the threatened species, *Cycladenia humilis* var. *jonesii*

**DOI:** 10.1093/conphys/cox053

**Published:** 2017-09-23

**Authors:** Valerie C Pence, Linda R Finke, Mary F Chaiken

**Affiliations:** 1 Center for Conservation and Research of Endangered Wildlife (CREW), Cincinnati Zoo & Botanical Garden, 3400 Vine Street, Cincinnati, OH 45220, USA

**Keywords:** Cryopreservation, endangered species, exceptional plant, germination, hyperhydricity, *in vitro*, micropropagation

## Abstract

*Ex situ* conservation is critical for hedging against the loss of plant diversity. For those species (exceptional species) that cannot be conserved long-term in standard seed banks, alternative methods are required, often involving *in vitro* culture and cryopreservation, or storage in liquid nitrogen. *Cycladenia humilis* var. *jonesii* is a federally threatened perennial native to Utah and Arizona. It is classified as an exceptional species, because it produces few seeds, and, thus, *in vitro* propagation and cryopreservation were investigated as tools for its propagation and preservation. Shoot-propagating cultures were established from both seedling and wild-collected shoots, but cultures from both sources displayed an extreme form of the physiological disorder, hyperhydricity. This phenotype could be at least partially normalized by the use of vented closures, as well as by using agar, rather than gellan gum, in the medium. The hyperhydric (HH) phenotype had a lower dry weight, more branching, minimal leaf development and more poorly developed vascular tissue than the more normal (MN) phenotype. Only more normalized shoots could be rooted and the resulting plants acclimatized. Both HH and MN shoots also provided shoot tips capable of surviving cryopreservation using the droplet vitrification method. These *in vitro* and cryopreservation methods provide tools that can be used for propagating plants of *C. humilis* var. *jonesii* for research and restoration, as well as for supplying shoot tips for the *ex situ* conservation of this species. The two distinct phenotypes also provide a useful system for studying factors involved in the HH response of this dryland species *in vitro*.

## Teaser

This study developed tissue culture and cryopreservation methods for the threatened *Cycladenia humilis* var. *jonesii* as tools for propagation and *ex situ* conservation, since it produces few seeds for banking. The cultures initially displayed abnormal (hyperhydric) growth, but more normal characteristics were promoted by venting the cultures.

## Introduction

*Ex situ* conservation is increasingly important as plant diversity is threatened by habitat loss, unsustainable use and climate change (Corlett, 2016). Whereas for most species, seed banking is the most effective and efficient method for conserving plant diversity long-term, it is not applicable to all species. Some seeds are intolerant of or short-lived in the dry, freezing conditions (15% RH, −20°C) used in current seed banking (FAO, 2014), while other species produce inadequate or no seed for banking. For these species, known as *exceptional species*, alternative methods are required for long-term germplasm storage, and such methods are highly dependent on techniques of *in vitro* culture and liquid nitrogen (LN) storage, or cryopreservation (Pence, 2013). These approaches have been demonstrated to be effective for a number of economically important species (Reed, 2008) and are beginning to be more widely applied to species of conservation concern (Pence, 2014).

*Cycladenia humilis* Benth. var. *jonesii* (Eastw.) S.L. Welsh & N.D. Atwood (Apocynaceae) is a federally threatened dryland perennial found at only five locations in the Canyonlands region of Utah and Arizona in the western U.S. and is represented by a little more than 1000 genetically distinct individuals in the wild (U.S. Fish & Wildlife Service, 2008). Threats include oil drilling, mining, and tar sands activities, as well as mountain bike and off-road vehicle recreation. Although it is a rare, monotypic genus, which has been little studied, it is in a family known for producing pharmacologically important chemicals, such as the anti-hypertensive reserpine and the anti-cancer vincristin and vinblastin (Dey and De, 2010; Pan *et al.*, 2016). *Cycladenia humilis* var. *jonesii* is also highly adapted to a dry, desert habitat. Thus, it is a species of significant potential interest for studies of drought tolerance, production of biologically active chemicals and other lines of investigation.

*Cycladenia humilis* var. *jonesii* produces few seeds, has very low seed viability and the seeds are difficult to germinate, while pollinators and seedlings have not been observed in the wild (Sipes *et al.*, 1994; Sipes and Tepedino, 1996). Thus, in addition to posing challenges for propagation, it is an example of an exceptional species. As a result, vegetative-based methods of propagation and preservation are of interest, including micropropagation, in order to provide material for research, *ex situ* conservation and potential restoration. This study investigated the potential for initiating shoot-propagating cultures from both seedlings and wild-collected shoot tips and evaluated some of the factors influencing the ability of those cultures to produce normal plants and to provide tissues for successful cryopreservation.

## Materials and methods

Unless otherwise indicated, chemicals used in this study were from Sigma-Aldrich Chemical Co., and prepared powders of basal medium salts were from PhytoTechnology Laboratories.

Wild-collected seeds of *C. humilis* var. *jonesii*, hereafter referred to as *C. humilis*, were received at the Center for Conservation of Endangered Wildlife (CREW) from Red Butte Garden and Arboretum, Salt Lake City, UT, including 30 seeds from 4 maternal lines, 3, 4, 6 and 17 seeds, respectively. These were surface sterilized with a 1:10 dilution of freshly opened commercial bleach (Austin’s A-1, 5.25% sodium hypchlorite) plus 0.05% Tween 20 for 10 min, rinsed in sterile, purified water, and placed onto sterile 60 × 15 mm disposable petri plates with 0.8% agar plus 100 mg/l of the fungicide, benlate (methyl 1-(butylcarbamoyl)-2-benzimidazolecarbamate), with approximately 15 ml of medium per plate. The plates were incubated at 4°C for 2 months and then moved to constant 26°C with a 16:8 h light:dark cycle under cool white fluorescent lights, at approximately 20–30 μmol/m^2^/s photosynthetically active radiation (PAR). When germination did not occur within 2 weeks, the seeds were scarified by nicking with a scalpel, moved to plates of a low nutrient medium of half-strength Murashige and Skoog (Murashige and Skoog, 1962) (MS) medium plus 1.5% sucrose gelled with 0.33% Gelzan (Caisson Labs) with 100 mg/l benlate. After 2 months, 1 seed had germinated. The remaining seeds were returned to 4°C for a second stratification for 2 months and then placed in an alternating temperature incubator (Precision Scientific 818), with 16:8 h of 25°C-light/15°C-dark, under cool white fluorescent lights, at approximately 30–40 μmol/m^2^/s PAR. The resulting seedlings were used to initiate shoot cultures.

Shoot cultures were initially grown on media that were based on full-strength MS salts (MS medium), but preliminary studies indicated that the shoots showed more growth on the salt formulation of Driver and Kuniyuki (Driver and Kuniyuki, 1984) with MS (Murashige and Skoog, 1962) organics (DKW medium). Initial observations also indicated that, while shoots that were grown on medium gelled with 0.8% agar appeared slightly less hyperhydric (HH) than those on 0.25% gel, when they were transferred from the agar medium to either agar or gel, the resulting growth was poor, with more browning compared with shoots transferred from gel. Thus, stock cultures were maintained on DKW medium, 3% sucrose, 0.5 mg/l BAP and 0.25% gel (maintenance medium, MM), in culture tubes (15 ml/tube) and in Magenta boxes (60 ml/box) with solid (unvented) closures. Cultures were held at 26°C and a 16:8 h light:dark cycle under fluorescent lights at approximately 45 μmol/m^2^/s PAR. For further work, only one line of the seedling-sourced material was maintained and designated Chj-1. One wild-collected shoot as well as a number of leaves were received from Red Butte Garden and Arboretum. Most of the leaves were used for dry weight determinations. The shoot was surface sterilized as for the seeds. The shoot was cut into six nodal stem segments that were placed onto MM plus benlate, and one segment gave rise to line Chj-2, which was maintained as for Chj-1.

**Figure 1:**
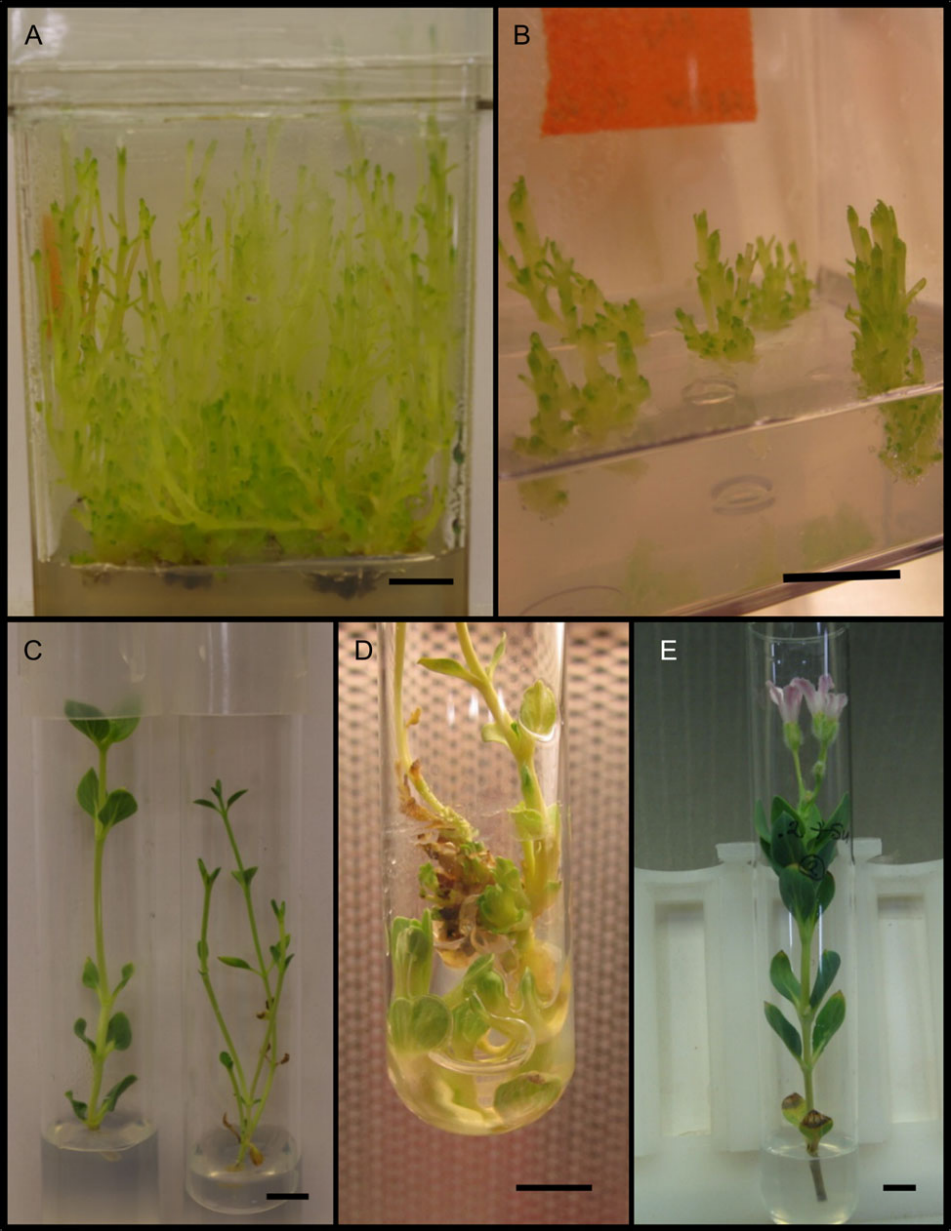
Shoot stages and phenotypes in this study. (**A**) HH shoots of Chj-1 at 4 weeks, grown in unvented boxes; (**B**) Shoot cluster bases of Chj-1 used as initial explants for the venting/gelling agent study, taken from HH cultures; (**C**) MN phenotypes of Chj-1 (left) and Chj-2 (right); (**D**) Stubby phenotype of Chj-1; (**E**) MN shoot of Chj-1 flowering *in vitro.* Bars = 1 cm

The effects of venting and gelling agent on shoot growth and development were examined by culturing shoots in boxes of MM with agar or gel, with and without venting. Explants for this experiment were created from clumps of HH shoots from stock cultures by cutting back stems that were greater than 1 cm in height, trimming away any basal callus and using the base tissue which contained masses of HH shoots (Fig. 1A and B). Five clumps per box or one per tube were used. Agar at 0.8% and gel at 0.25% were used to compare gelling agents.

To create vented lids for Magenta GA-7 boxes, four 7 mm diameter holes were made using a heated cork borer, and these were covered with 3M Micropore™ surgical tape. Vented caps for culture tubes were produced by using Magenta™ 2-Way Caps in which the top surface had cracked. The cracked area was removed to create a circular opening approximately 18 mm in diameter and this was similarly covered with surgical tape. Vented lids were autoclaved separately from the medium and placed onto the containers only after the tissue was added to the medium, to minimize drying of the medium during storage at 4°C before use.

The HH and more normal (MN) phenotypes were evaluated in several ways. Tissue dry weight was determined gravimetrically and calculated on a wet weight basis (1-(WW-DW/WW)) for 10 samples per treatment. Dry weight was also determined for 42 samples from wild-collected leaves. The extent of branching was measured as the number of shoot tips on shoots >2.5 cm, with *n* ranging from 23 to 32 per treatment. Vessel development was observed in hand-cut cross sections of HH and MN stems using a phloroglucinol/HCl/ethanol reagent stain of 2 g phloroglucinol dissolved in 80 ml of 20% ethanol with 20 ml of concentrated HCl added. Development of stomata was analyzed by microscopic observation of leaf impressions made in clear fingernail polish.

For rooting, MN shoots grown for one or two subcultures on DKW medium with 0.5 mg/l BAP with gel and venting in tubes were transferred to DKW medium with 1 mg/l indolebutyric acid (IBA), 0.8% agar, with venting in tubes. Rooted shoots were acclimatized in non-sterile soil boxes, consisting of a 1:1:1 mix of pea gravel (Quikrete Pea Pebbles):sand (Quikrete Premium Play Sand):Pro-Mix (Pro-Mix BX Mycorrhizae™, Premier Horticulture, Inc.) in covered plastic boxes (Sigma Phytatray II) with 5, 3 mm diameter holes made in the lids with a hot nail, and maintained at room temperature in the laboratory (21–23°C) under cool white fluorescent lights with a 16:8 h light:dark cycle, 80–90 umol/m^2^/s PAR.

For cryopreservation, shoot tips approximately 1–2 mm in length were isolated from both Chj-1 and Chj-2 lines and pre-cultured for 1 day on plates of MS medium with 3% sucrose, 0.1 mg/l BAP, 0.01 naphthaleneacetic acid (NAA), 10 μM abscisic acid, 0.3 M mannitol and 0.25% gel. They were cryopreserved using the droplet vitrification method (Panis *et al.*, 2005), using the following cryoprotective steps: 20 min in a loading solution (MS salts, 0.4 M sucrose, 2 M glycerol) at room temperature followed by 20 min in cold PVS2 (MS salts, 0.4 M sucrose, 30% glycerol, 15% ethylene glycol and 15% DMSO) and held on ice. Some tips were moved to rinsing solution and then to recovery medium (see below) as a PVS2 control, while the remaining tips were transferred to a sterile strip of aluminium foil, approximately 0.8 × 3.0 cm with a small amount of PVS2. The foil and tips in PVS2 were rapidly exposed to LN and then moved to an LN cooled empty cryovial and maintained in LN for 1 h. Rapid rewarming of the shoot tips was accomplished by immersion of the foil strip and tips in rinsing solution (MS salts and 1.2 M sucrose) at room temperature, where the tips remained for 15 min before transfer to recovery plates of MS medium with 0.5 mg/l BAP and 0.05 or 0.5 mg/l NAA (designated 0.5B 0.05N and 0.5B 0.5N media, respectively), 3% sucrose and 0.25% gel. Survival, the production of callus, and the outgrowth of shoots were recorded at 1, 2 and 4 weeks, with further shoot development followed for 5 samples for an additional 1–2 months. Each experiment used 10–15 tips per treatment (PVS2 control and LN exposed); one experiment was done using the HH phenotype of Chj-1, while all other experiments with Chj-1 and Chj-2 were done using the MN phenotype.

Data were analyzed and graphed using JMP® Version 10 (SAS Institute Inc.). Both dry weight and branching were analyzed by a one-way ANOVA and a means comparison using the Tukey *post hoc* test.

## Results

### Culture initiation from seeds and wild-collected tissues

After the application of stratification, scarification, a second stratification and exposure to alternating temperatures, a total of 9 of the 30 seeds germinated (30%). Of the 4 maternal lines, germination rates were 0%, 17%, 25% and 41%, with the highest rate being in the line that had the highest number of seeds. Shoots from all 9 seedlings appeared HH and maintained that phenotype when used to initiate shoot-propagating cultures, although only one genotype, Chj-1, was maintained for subsequent experiments. About three of the six nodal stem segments cultured from the wild-collected shoot became contaminated with bacteria, but all of the remaining three showed outgrowth of the lateral buds. All of these were HH on MM, and one line was maintained further as Chj-2.

### Developing and propagating the MN phenotype

Preliminary tests indicated that when cultures were vented, the HH phenotype began to show a MN phenotype, and with successive transfers, the shoots of both Chj-1 and Chj-2 showed progressively MN features, including more opaque shoots and broad, flattened leaves, particularly as the shoots grew toward the vented cap. There were readily observable differences in leaf shape between the two lines, as well as more frequent formation of axillary shoots in the Chj-2 line (Fig. 1C). MN shoots could be maintained by transferring the apical stem segment to fresh medium, but, because branching was rare, particularly in Chj-1 even in decapitated shoots, they did not readily multiply. When branching did occur, only one of the two nodal buds developed, and development of shoots from isolated nodal sections did not readily occur. Occasionally, although not predictably, a cluster of dense tissue generating numerous buds and shoots appeared from MN shoots, initiated from a node below the medium surface (Fig. 1D). These shoots had thicker, more turgid (‘stubby’) stems and smaller leaves than other MN shoots, but could be grown into robust MN shoots. The numerous basal buds could be sub-cultured to quickly generate additional MN shoots. Also occasionally, flower buds developed, in addition to the vegetative buds in the MN shoots, and rarely these developed into flowers (Fig. 1E).

### Characterization of the HH and normal phenotypes

**Figure 2:**
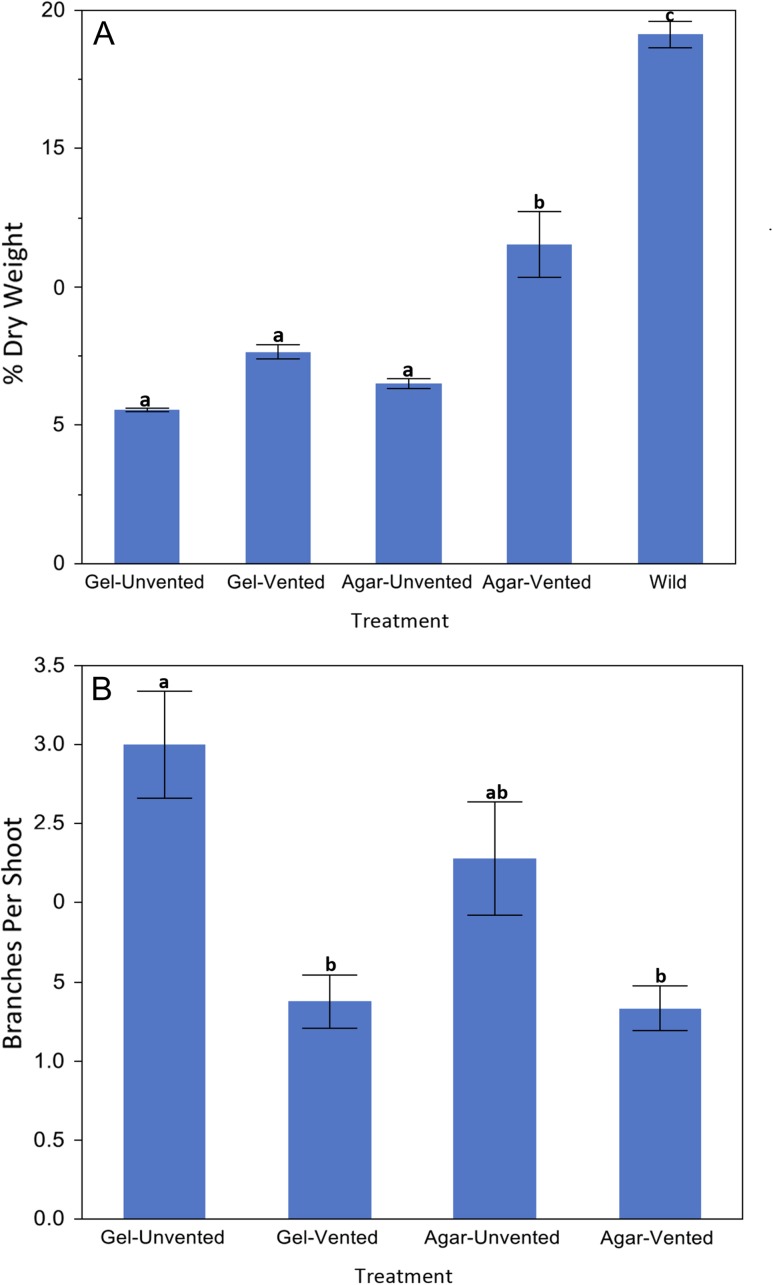
Effects on dry weight (**A**) and branching (**B**) of shoots of Chj-1 grown for 4 weeks in Magenta boxes on DKW medium with 0.5 mg/l BAP with and without venting, with agar or gel (*n* = 10 per treatment). DW was also compared with that of wild tissues (*n* = 42). Different letters designate significant differences (Tukey, *P* < 0.05)

**Figure 3:**
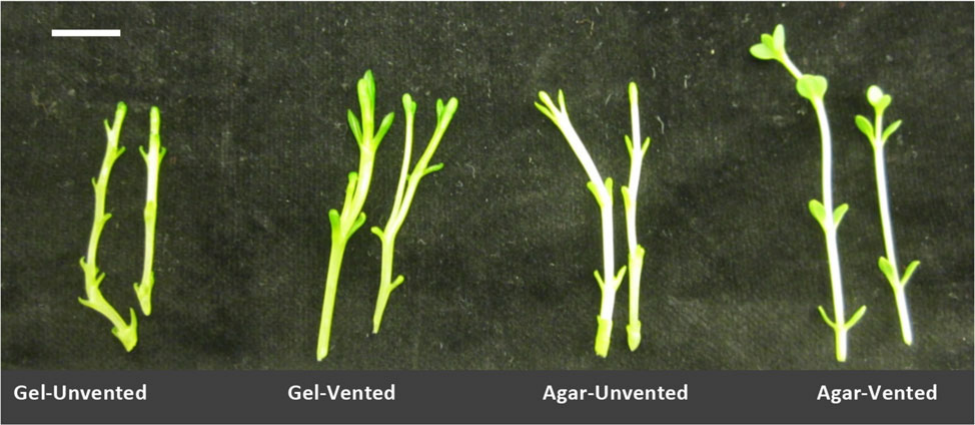
Effect of venting and gelling agent on leaf development of Chj-1 grown for 4 weeks in Magenta boxes on DKW medium with 0.5 mg/l BAP. Bar = 1 cm

**Figure 4:**
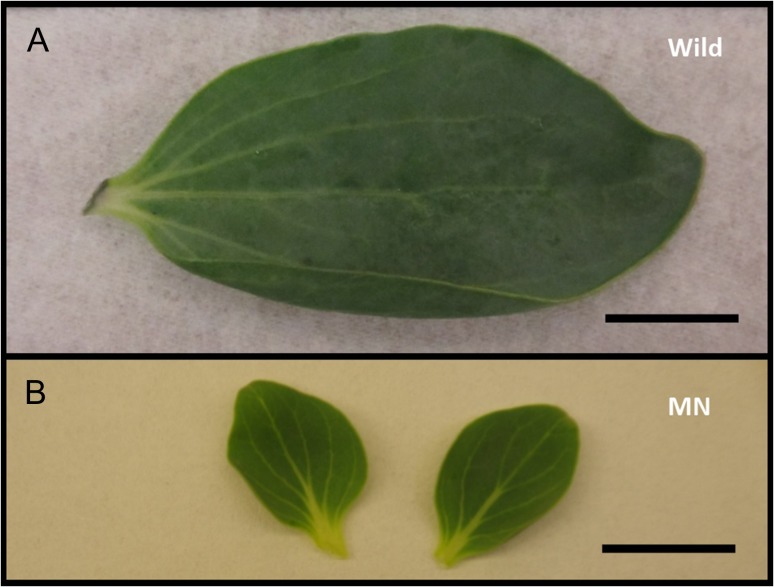
(**A**) Leaf collected from a plant in the wild. (**B**) Leaves from MN shoots of Chj-1. Bars = 1 cm

**Figure 5:**
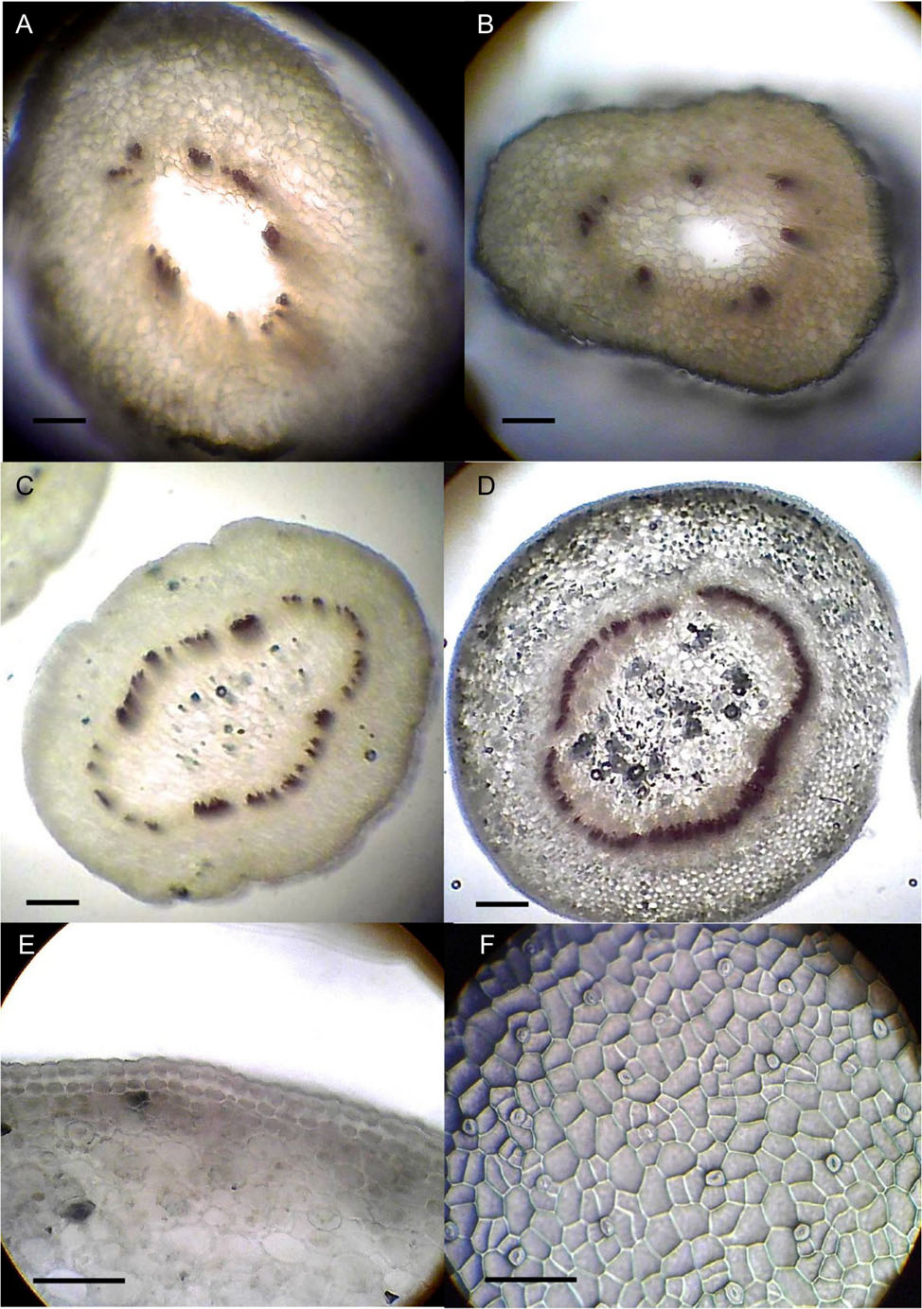
Stem and leaf development in HH and MN shoots of Chj-1. Stem cross sections (A–E): (**A**) HH stem; (**B**) intermediate stem; (**C**) MN stem after one subculture with vents; (**D**) stem normalized through several subcultures with vents; (**E**) stem normalized through several subcultures with vents, showing epidermal and collenchyma layers; (**F**) impression of leaf surface of leaf normalized through several subcultures with vents. Bar = 0.2 mm (A–D), 0.1 mm (E, F)

With the generation of a MN phenotype for this species, HH and MN tissues of line Chj-1 were compared for several characteristics, and these were, in turn, compared with the small amount of wild tissue that became available during the course of this study. Shoots taken from HH cultures and transferred to four combinations of agar/gel × vented/unvented for one subculture had a significantly higher percent dry weight on the agar/vented treatment compared with the other cultures (Fig. 2A), although this was still significantly lower than the average dry weight of wild tissues (*F*_4_ = 103.1, *P* < 0.0001). In the same experiment, the HH cultures on gel/unvented had significantly more branching than vented cultures on either gel or agar (*F*_3_ = 9.0, *P* < 0.0001) (Fig. 2B). In the same experiment, stems on the unvented gel medium appeared the most HH, with translucent green and brittle stems and short internodes, with very little leaf development, while the MN stems, on the vented agar medium, were more opaque, paler green and rubbery, with elongated internodes and more developed leaves (Fig. 3). As shoots were put through several sequential transfers in vented containers, they became MN appearing (Fig. 1C), with larger, flattened, more broadly obovate leaves with palmate venation. While these leaves were somewhat smaller than wild-collected leaves, they were similar in shape, texture and venation pattern (Fig. 4). Xylem tissue of HH stems was poorly developed and occurred in sparse, disjointed bundles (Fig. 5A). Partially normalized tissues still showed few and disjunct bundles (Fig. 5B), while MN stems possessed a fully formed ring of vascular tissue and a well-formed epidermis and collenchyma region (Fig 5C–E). Stomata could not be evaluated on HH leaves, since development of the leaf was minimal. However, leaves of MN shoots showed a regular distribution of well-formed stomata on both upper and lower surfaces (Fig. 5F).

### Rooting and acclimatization

**Figure 6:**
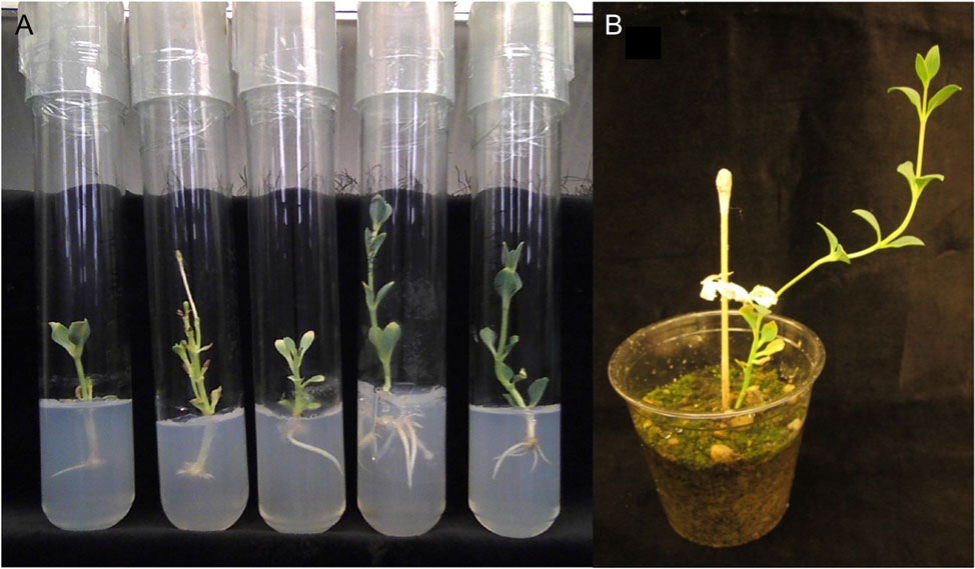
(**A**) Shoots of Chj-1 rooted *in vitro* on medium with 1 mg/l IBA; (**B**) rooted shoot acclimatized in soil

HH shoots of *C. humilis* did not form roots, either on MM or when cultured on medium containing IBA. However, shoots taken from MN cultures formed roots (Fig. 6A) at a rate of up to 33%, depending on the trial. Several rooted shoots were acclimatized in soil, with survival of up to 9 months, where the shoots continued to grow without branching (Fig. 6B).

### Cryopreservation of shoot tips

**Figure 7:**
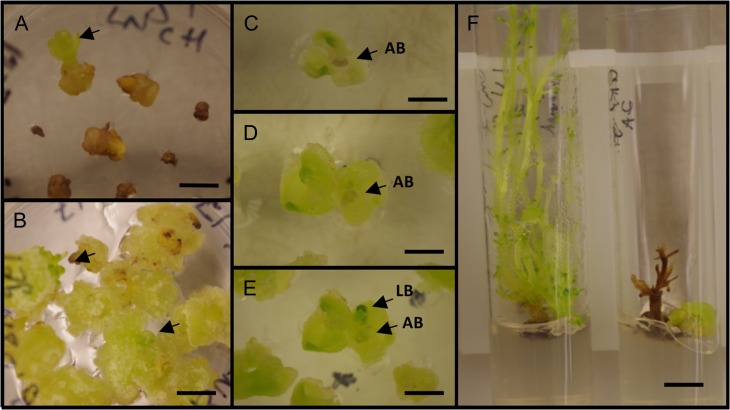
LN exposed shoot tips of Chj-1 recovered (**A**) on 0.5B 0.05 N medium, showing arrested growth in most tips and one shoot elongating (arrow), at 6 weeks; and (**B**) on 0.5B 0.5 N medium and transferred to 0.5B 0.05N medium, 2 weeks after thawing, showing callus and beginning shoots (arrows) at 6 weeks; (**C**–**E**) LN exposed shoot tip of Chj-1 recovered on 0.5B 0.5N medium at 1, 2 and 3 weeks, respectively, with transfer to 0.5B 0.05N medium at 2 weeks, showing the browned apical bud (AB) and the outgrowth of a lateral bud (LB); (**F**) growth from LN exposed shoot tips of Chj-2, showing continued growth into HH shoots (left) and arrested growth (right), at 2 months. Bars A, B = 0.5 cm; C—E = 0.25 cm; F = 1 cm

Shoot tips were isolated from the MN phenotype of both Chj-1 and Chj-2 and were subjected to the droplet vitrification protocol. Growth after exposure to PVS2 or PVS2 and LN was strongly influenced by the level of NAA in the recovery medium (Table 1). When recovered on medium with 0.5 mg/l BAP and 0.05 mg/l NAA, both PVS2 and LN exposed shoot tips showed high survival in terms of enlargement with little or no callus, but after 2–3 weeks, most of these tips browned and did not grow further (Fig. 7A). With Chj-1, some shoots showed early outgrowth on this medium, but this was not observed with Chj-2 shoot tips, and more development was observed in the PVS2 control than with the LN exposed tips. When recovered on medium in which NAA was increased 10-fold to 0.5 mg/l, almost all shoot tips of both the PVS2 control and the LN treated samples remained green longer than on the lower auxin level. They also developed callus from most of the cut surfaces (Table 1; Fig. 7B), often initiating outgrowth of shoots as well, although more shoots were initiated from PVS2 treated than from LN exposed shoot tips. Transfer of the tissues from 0.5B 0.5N to 0.5B.05N medium after 1 or 2 weeks did not appear to increase the number of lateral buds growing out as shoots (data not shown). In some cases, browning of the apical bud was obvious after LN exposure (Fig. 7C), but other areas of the shoot remained green, formed callus and outgrowth of lateral buds occurred (Fig. 7D and E), However, in the 5 shoot-forming treatments where shoot growth was followed longer than 1 month, the further outgrowth of the shoots was variable, with an average of only 18% of shoots continuing to grow enough to establish a shoot-propagating culture, while the remaining shoots became arrested in their development (Fig. 7F). Preliminary results using shoot tips from HH cultures grown on MM in unvented cultures and cryopreserved using the same protocol and sample sizes as for the MN shoot tips, indicated shoot production at rates of 83% and 92% for PVS2 controls and 100% and 92% for LN exposed shoot tips after 1 month on the lower and higher levels of NAA, respectively.

**Table 1:** Percent survival of shoot tips of the two genotypes of *C. humilis* through PVS2 and LN exposure using the droplet vitrification procedure and recovered on two different media

Line	Recovery medium	No.^a^	PVS2				LN		
			Alive^b^	Callus^c^	Shoots^d^	No. ^a^	Alive^b^	Callus^c^	Shoots^d^
**Chj-1**	**0.5B.05N**	**3**	**100**	**0**	**53.3 ± 18.6**	**4**	**88.6 ± 5.6**	**0**	**2.5 ± 2.5**
	**0.5B.5N**	**4**	**100**	**100**	**90 ± 5.8**	**5**	**96.7 ± 2.1**	**86.7 ± 6.9**	**10.7 ± 4.5**
**Chj-2**	**0.5B.05N**	**2**	**100**	**0**	**0**	**2**	**90 ± 10**	**0**	**0**
	**0.5B.5N**	**2**	**100**	**100**	**100**	**2**	**96.7 ± 3.3**	**96.7 ± 3.3**	**54 ± 0.5**

^a^No. = number of replicate experiments; 10–15 shoot tips were used per treatment in each replicate experiment.

^b^Alive = percent remaining green with some swelling after at least 1 week on recovery medium.

^c^Callus = percent producing callus from the cut surfaces within the first 2 weeks on recovery medium.

^d^Shoots = percent producing some outgrowth of apical or lateral shoots within the first month on recovery medium.

## Discussion

These studies have demonstrated that both *in vitro* and cryopreservation methods can be applied to the propagation and preservation of the rare, dryland species, *C. humilis* var. *jonesii*, both from seedling material and from wild-collected shoots. Seed germination in this species has been reported as being difficult and seeds are not abundantly produced (U.S. Fish and Wildlife Service, 2008). Our studies achieved 30% germination overall and over 40% in one maternal line using a combination of scarification, stratification, and alternating temperatures for germination. However, with the limited seed available, these dormancy-breaking treatments could not be evaluated individually. A number of herbaceous desert perennials exhibit physiological dormancy (Baskin and Baskin, 1998), and additional studies with *C. humilis* could increase germination above the level seen in our studies. However, as seeds are not frequent in this species, the ability to initiate cultures from small amounts of vegetative material, as was done in this study, provides a more reliable source of material for propagation. Even though one line each was selected from the seedling and wild-collected materials for further study, all shoots initiated from seedlings and the wild-collected shoot grew with a similar HH phenotype *in vitro* when initiated on gel and in closed containers.

The hyperhydricity observed in *C. humilis* responded to venting in a manner similar to that observed in several other species (Dantas *et al.*, 2001; Park *et al.*, 2004; Lai *et al.*, 2005; Badr-elden *et al.*, 2012; Reyes-Vera *et al.*, 2008). The effects of venting may be due to a reduction in humidity levels, as it has been hypothesized that high humidity in tissue culture systems may lead to waterlogging of the apoplast in the plant tissues, which, in turn, can give rise to hypoxia and stress (Van Den Dries *et al.*, 2013). Other potential sources of stress resulting in hyperhydricity include high water availability from the medium, as contributed by gel compared with agar, high levels of nitrogen and high concentrations of cytokinin in the medium (Gaspar *et al.*, 2000, 1995; Franck *et al.*, 2004; Kevers *et al.*, 2004; Ivanova and Van Staden, 2011). Venting may also act by reducing the levels of ethylene in the culture headspace (Zobayed *et al.*, 1999), as ethylene is commonly produced by plant tissues under stress (Wang *et al.*, 2013) and has been correlated with some of the abnormal growth symptoms observed in HH tissues (Fal *et al.*, 1999; Park *et al.*, 2004). The stress of hyperhydricity has been correlated with elevated levels of factors associated with protection against reactive oxygen species, and preliminary studies in this lab also suggest that MN tissues had higher protein levels and lower catalase activity than HH tissues.

The HH phenotype of *C. humilis* has many of the characteristics described for other HH species, including brittle, glassy stems, poor leaf development and poor vascular development (Torre *et al.*, 2003; Kevers *et al.*, 2004; Fauguel *et al.*, 2008; Jausoro *et al.*, 2010; Picoli *et al.*, 2008). Whereas in many species abnormal leaves develop in HH cultures, in *C. humilis* leaf development is almost entirely inhibited, with little or no enlargement of the lamina. HH tissues of *C. humilis* had lower percent dry weights compared with MN tissue, reflecting higher water content, a general characteristic of HH tissues (Crevecoeur *et al.*, 1987; Chakrabarty *et al.*, 2005).

The improved rooting response with the MN phenotype in *C. humilis* is similar to that reported for *Atriplex canescens* after alleviation of hyperhydricity in that species (Reyes-Vera *et al.*, 2008). However, the fact that only about one-third of *C. humilis* shoots formed roots in the presence of auxin suggests that the shoots are not entirely normalized or that the medium and conditions are not yet optimized for root formation. Medium salts have been shown to be important for rooting (Oberschelp and Gonçalves, 2016), and future work with *C. humilis* should examine factors in addition to venting and gelling agent that might further normalize *in vitro* shoots and improve their ability to produce roots.

While a number of species have exhibited hyperhydricity *in vitro*, the extreme form of hyperhydricity exhibited by *C. humilis*, characterized by an almost total lack of leaf expansion, combined with its ability to form the MN phenotype, suggest that this species could provide a unique tool in the study of hyperhydricity, a physiological disorder that can inhibit the production of normal plants for commerce and conservation (Hazarika, 2006). As a wild, dryland species, it is likely to provide insights into hyperhydricity and plant stress that are different from highly selected, economically important species. Questions regarding stress responses to changes in water and nitrogen availability are becoming especially relevant in the face of climate change. As some deserts are predicted to become wetter in the future, while others will become drier (Donat *et al.*, 2013) , dryland species such as *C. humilis* should provide useful models for understanding plant adaptations to extreme environments and their ability to adapt to change.

A more immediate outcome of this work is a protocol for propagating this rare species in spite of poor seed production, and the potential for generating larger numbers of plants for restoration and research. In addition, this work has shown that *in vitro* cultures can provide material for shoot tip cryopreservation (Reed, 2008). Such shoot tips can be used for long-term germplasm conservation, given the low number of seeds available for seed banking. Survival and growth of shoot tips through LN exposure was high, although continued growth was dependent on the presence of auxin, which appeared to also stimulate significant callus growth. In other systems, auxin has been eliminated from recovery media to reduce callus growth from shoot tips after cryopreservation (Chang and Reed, 1999), but *C. humilis* appears to have a requirement for at least some auxin in the recovery medium. Reduced NAA levels or the use of alternative auxins might reduce callus without altering growth in this system. In addition, while there have been reports of hyperhydricity in shoot tips growing after cryopreservation (Poisson *et al.*, 2016), to our knowledge, this is the first report of survival of HH shoot tips through cryopreservation. Because of the much higher level of branching in this phenotype of *C. humilis*, HH shoots are more easily generated than MN shoots and could reduce the time and resources needed for producing materials for long-term cryopreservation. While further improvements in recovery conditions should increase the rate of shoot growth after cryostorage, the methods described here provide the basis for the *ex situ* conservation of this exceptional species. Shoot tips from both the Chj-1 and Chj-2 lines of *C. humilis* are currently in long-term storage in CREW’s CryoBioBank.®

## Conclusions

Both *in vitro*-germinated seedling shoot tips and wild-collected shoot tips from new growth on mature plants can be used to initiate *in vitro* shoot cultures of the threatened dryland species, *C. humilis* var. *jonesii.* Such cultures exhibit an extreme form of hyperhydricity when grown on medium in unvented containers, but a MN phenotype can be developed by subculture in vented containers. Some rooting can be achieved from the partially normalized shoots, providing plants for acclimatization, but further work is needed to produce shoots that can be rooted with a higher rate of success. Shoot tips were successfully cryopreserved using the droplet vitrification method, showing good survival and some shoot production after LN exposure. These studies demonstrate that the tools of *in vitro* propagation and shoot tip cryopreservation can be applied to *C. humilis* and are available for the *ex situ* conservation and potential restoration of this species.
